# Comparison of diagnostic performance of four software packages for phase dyssynchrony analysis in gated myocardial perfusion SPECT

**DOI:** 10.1186/s13550-017-0274-3

**Published:** 2017-03-23

**Authors:** Koichi Okuda, Kenichi Nakajima, Shinro Matsuo, Soichiro Kashiwaya, Hiroto Yoneyama, Takayuki Shibutani, Masahisa Onoguchi, Mitsumasa Hashimoto, Seigo Kinuya

**Affiliations:** 1 Department of Physics, Kanazawa Medical University, 1-1 Daigaku, Uchinada, Kahoku, Ishikawa 920-0293 Japan; 2 Department of Nuclear Medicine, Kanazawa University Hospital, 13-1 Takara-machi, Kanazawa, Ishikawa 920-8641 Japan; 3 Department of Radiological Technology, Kanazawa Municipal Hospital, Kanazawa, Japan; 4 Department of Radiological Technology, Kanazawa University Hospital, Kanazawa, Japan; 5 Department of Quantum Medical Technology, Kanazawa University, Kanazawa, Japan

**Keywords:** Phase analysis, Left ventricular, Mechanical dyssynchrony, Myocardial perfusion SPECT, Cardiac resynchronization therapy

## Abstract

**Background:**

Phase analysis of gated myocardial perfusion single-photon emission computed tomography (SPECT) for assessment of left ventricular (LV) dyssynchrony was investigated using the following dedicated software packages: Corridor4DM (4DM), cardioREPO (cREPO), Emory Cardiac Toolbox (ECTb), and quantitative gated SPECT (QGS). The purpose of this study was to evaluate the normal values of 95% histogram bandwidth, phase standard deviation (SD), and entropy and to compare the diagnostic performance of the four software packages. A total of 122 patients with normal myocardial perfusion and cardiac function (58.9 ± 12.3 years, 60 women, ejection fraction (EF) 74.3 ± 5.7%, and end-diastolic volume (EDV) 83.5 ± 3.6 mL) and 34 patients with suspected LV dyssynchrony (64.1 ± 12.2 years, 9 women, EF 52.0 ± 18.0%, and EDV 145.0 ± 6.8 mL) who underwent Tc-99m methoxy-isobutyl-isonitrile/tetrofosmin gated SPECT were retrospectively evaluated. Dyssynchrony indices of the 95% histogram bandwidth, phase SD, and entropy were computed with the four software programs. Diagnostic performance of LV phase dyssynchrony assessments was determined by receiver operator characteristic (ROC) analysis. The area under the ROC curve (AUC) was used to compare the software programs. The optimal cutoff point was determined by ROC curve based on the Youden index.

**Results:**

The average of normal bandwidth significantly differed among the four software programs except in the comparison of 4DM and ECTb. Moreover, the normal phase SD significantly differed among the four software programs except in the comparison of cREPO and ECTb. The software programs showed high correlation levels for bandwidth, phase SD, and entropy (*r* ≥ 0.73, *p* < 0.001). ROC AUCs of bandwidth, phase SD, and entropy were ≥0.850, ≥0.858, and ≥0.900, respectively. Moreover, the ROC AUCs of bandwidth, phase SD, and entropy did not significantly differ among the four software programs. Optimal cutoff points for phase parameters were 24°–42° for bandwidth, 8.6°–15.3° for phase SD, and 31–48% for entropy.

**Conclusions:**

Although the optimal cutoff value for determining LV phase dyssynchrony by ROC analysis varied depending on the use of the different software programs, all software programs can be used reliably for phase dyssynchrony analysis.

## Background

Cardiac resynchronization therapy (CRT) has been shown to be beneficial for patients with severe heart failure (HF) who do not respond to treatment with medications. The three eligibility criteria for CRT are a New York Heart Association (NYHA) functional class III or IV, left ventricular ejection fraction (LVEF) ≤35%, and QRS duration >120 ms in cardiac echocardiography [1, 2]. Although patients with HF meet the eligibility criteria, one-third of patients did not respond to CRT [3, 4].

In order to improve the selection criteria for patients with HF to predict sufficient response to CRT, assessment of left ventricular (LV) mechanical dyssynchrony has been proposed. The LV mechanical dyssynchrony of patients with HF can be assessed using noninvasive imaging modalities such as an echocardiography with tissue Doppler imaging [3, 4] and a magnetic resonance imaging (MRI) [5, 6]. Nuclear cardiology techniques, i.e., gated myocardial perfusion single-photon emission computed tomography (SPECT) (GMPS), have also been used to diagnose LV mechanical dyssynchrony [7–10]. Quantitative assessment of LV mechanical dyssynchrony by GMPS can be performed using commercially available software programs, including Emory Cardiac Toolbox (ECTb; Syntermed, Atlanta, GA, USA) [11], quantitative gated SPECT (QGS; Cedars-Sinai Medical Center, Los Angeles, CA, USA) [12], and Corridor4DM (4DM; INVIA Medical Imaging Solutions, Ann Arbor, MI, USA) [13] as well as cardioREPO (cREPO; FUJIFILM RI Pharma, Tokyo, Japan, developed in collaboration with EXINI Diagnosis, Lund, Sweden, and Kanazawa University, Kanazawa, Japan) [14–18]. The phase histogram and its distribution of the whole and regional LV can be automatically analyzed with these software programs, and these data can be utilized to diagnose LV dyssynchrony.

The purpose of this study was to clarify the normal values of 95% histogram bandwidth, phase standard deviation (SD), and entropy and to compare the diagnostic performance of the following software programs in phase analysis for identifying LV phase dyssynchrony: 4DM, cREPO, ECTb, and QGS.

## Methods

### Subjects

A total of 156 patients, who underwent ^99m^Tc-sestamibi (MIBI) or ^99m^Tc-tetrofosmin GMPS were retrospectively enrolled. Of these 156 patients, 122 were diagnosed as having normal perfusion (summed stress score ≤3) and cardiac function (EF ≥ 50%). The data for these patients were included in a Japanese normal database generated by the Japanese Society of Nuclear Medicine working group (JSNM-WG). The patient selection criteria for generating the normal database have been summarized elsewhere [19–22]. The remaining 34 patients who underwent GMPS in Kanazawa University Hospital were indicated for the determination of CRT implantation (*n* = 15), screening for the left bundle branch block (BBB) (*n* = 15), right BBB (*n* = 3), and pulseless ventricular tachycardia (*n* = 1). Of 34 patients, 15 patients were diagnosed as having heart failure with NYHA class III or IV symptoms. These clinical diagnoses were made separately in the Department of Cardiology. Consequently, we used these clinical diagnoses for discrimination between normal and abnormal phase distributions in GMPS data. The patient characteristics are shown in Table 1. Informed consent was obtained from all patients in the hospital. The ethics committee of Kanazawa University approved this study.

**Table 1:** Characteristics of the study population

	Patients with		*P* value
	Normal perfusion and cardiac function	Suspected LV dyssynchrony	
Men/women	62/60	25/9	n.s.
Mean age (y)	58.9 ± 12.3	64.1 ± 12.2	0.029
Height (cm)	161 ± 8.1	160 ± 8.8	n.s.
Weight (kg)	58.8 ± 10.4	60.9 ± 12.1	n.s.
BMI (kg/m^2^)	22.5 ± 3.0	23.7 ± 3.9	n.s.
BSA (m^2^)	1.61 ± 0.16	1.63 ± 0.18	n.s.
LV function			
LVEF (%)	74.3 ± 5.7	52.0 ± 18.0	<0.0001
LVEDV (mL)	83.5 ± 3.6	145.0 ± 6.8	<0.0001
LVESV (mL)	21.5 ± 6.7	79.7 ± 68.1	<0.0001
LV phase distribution			
Bandwidth (°)	38.4 ± 10.4	114.0 ± 84.6	<0.0001
Phase SD (°)	9.7 ± 0.88	27.7 ± 1.7	<0.0001
Entropy (%)	41.9 ± 6.2	63.2 ± 16.2	<0.0001
Myocardial perfusion SRS	0.6 ± 1.2	7.9 ± 9.5	<0.0001

LVEF, LVEDV, LVESV, bandwidth, phase SD, and entropy were calculated with a cardioREPO software program. SRS was calculated with a quantitative perfusion score (QPS) software program

Abbreviations: *BMI* body mass index, *BSA* body surface area by the DuBois formula, *LV* left ventricular, *EF* ejection fraction, *EDV* end-diastolic volume, *ESV* end-systolic volume, *SD* standard deviation, *SRS* summed rest score

### Image acquisition

With regard to the image acquisition condition for patients with suspected LV dyssynchrony, GMPS was performed with a dual-head gamma camera (Symbia T6 hybrid SPECT/CT scanner, Siemens Japan, Tokyo, Japan) equipped with a low-energy high-resolution (LEHR) collimator. A photopeak window of ^99m^Tc was set as a 15% energy window centered at 140 keV. The acquisition pixel size was 6.6 mm in a 64 × 64 matrix. Sixteen frames per cardiac cycle were used during data acquisition in GMPS. A circular orbit of the gamma cameras was adopted for 360° SPECT image acquisition with 60 projections. ^99m^Tc-MIBI or ^99m^Tc-tetrofosmin of 300–370 MBq was injected intravenously. The time per view of the SPECT acquisitions were 35 s (360° acquisition with 60 views), 50 s (180° acquisition with 32 views), and 60 s (180° acquisition with 30 views). The detailed acquisition conditions for the JSNM-WG normal database have been summarized elsewhere [19–22].

### Image analysis

The LVEF, end-diastolic volume (LVEDV), and end-systolic volume (LVESV) were automatically calculated with a cREPO software program. A histogram of phase distribution in the whole LV was computed from rest GMPS data. The phase SD and bandwidth were calculated with 4DM, cREPO, ECTb, and QGS programs. The bandwidth was expressed as 95% width of the phase histogram [7]. Entropy of the histogram distribution was calculated with QGS and cREPO because of unavailability in 4DM and ECTb [12, 19, 23]. Summed rest score (SRS) was calculated with a quantitative perfusion score (QPS; Cedars-Sinai Medical Center, Los Angeles, CA, USA) software program [12].

### Statistical analysis

All continuous values are expressed as a mean ± standard deviation. Student *t* test was used to analyze the differences among continuous variables. For the paired continuous variables, a paired *t* test was used to analyze the differences. Linear regression analysis was used to explain the relationship between two continuous variables. The Tukey-Kramer method was used for multiple comparisons of the bandwidth and phase SD among software programs. The Bland-Altman analysis was used for the assessment of the agreement. The receiver operating characteristic (ROC) area under the curve (AUC) values were expressed as mean ± standard error of the mean. Subsequently, the optimal cutoff points were determined by ROC curve based on the Youden index [24]. All statistical tests were two-tailed, and the *p* values ≤0.05 were considered significant. These analyses were performed using MedCalc version 14.12.0 (MedCalc Software bvba, Ostend, Belgium) and JMP version 11.2.1 (SAS Institute Inc., Cary, NC, USA).

## Results

### Study population

The mean age of patients with suspected LV dyssynchrony was slightly higher than that of those with normal perfusion (64.1 vs. 58.9 years, *p* = 0.029). With regard to LV function and phase distribution, the LVEF significantly decreased, and LVEDV, LVESV, bandwidth, phase SD, and entropy significantly increased in patients with suspected LV dyssynchrony in comparison with normal patients (*p* < 0.0001). The patients with suspected LV phase dyssynchrony had moderate myocardial perfusion abnormalities (mean SRS; 7.9 ± 9.5). When we calculated LVEF values in 15 patients with HF and 19 patients with echocardiographic abnormality, mean EF values were 38 ± 14% for those with HF and 64 ± 11% for those with electrocardiographic abnormality (*p* < 0.0001). Thus, this result showed that the patients with suspected LV dyssynchrony had slight to severe cardiac function abnormalities.

### Phase distribution

**Fig. 1:**
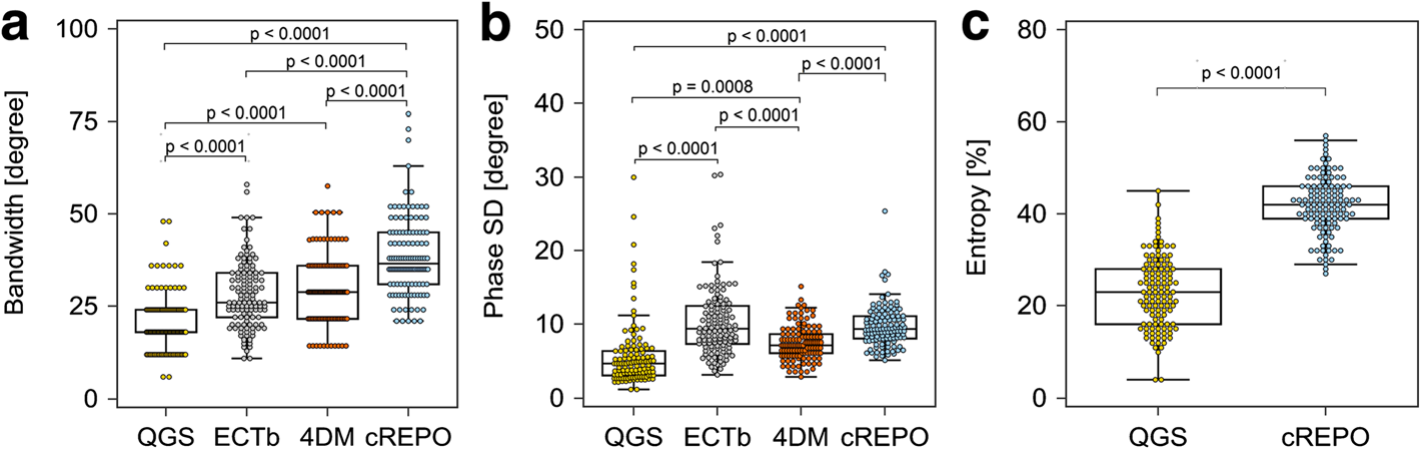
The box-and-whisker plots of bandwidth (**a**), phase SD (**b**), and entropy (**c**) in patients with normal perfusion and cardiac function (*n* = 122). These phase parameters were computed with QGS, ECTb, 4DM, and cREPO. *SD* standard deviation, *QGS* quantitative gated SPECT, *ECTb* Emory Cardiac Toolbox, *4DM* Corridor 4DM, *cREPO* cardioREPO

The phase distribution of patients with normal perfusion is shown in Fig. 1. The mean bandwidth significantly differed among the four software programs (QGS, 20.5° ± 7.8°; ECTb, 28.1° ± 9.1°; 4DM, 29.6° ± 9.3°; and cREPO, 38.4° ± 10.4°; *p* < 0.0001 for all combinations except the combination of ECTb and 4DM (*p* = n. s.)). The mean phase SD also significantly differed among the four software programs (QGS, 5.7° ± 4.4°; ECTb, 10.4° ± 4.8°; 4DM, 7.5° ± 2.3°; and cREPO, 9.7° ± 2.8°; *p* < 0.0001 for all combinations except the combination of QGS and 4DM (*p* = 0.008), and ECTb and cREPO (*p* = n. s.)). A significant difference was observed between the mean entropies calculated by QGS and cREPO (23.0 ± 7.7% vs. 41.9 ± 6.2%, *p* < 0.0001).

**Fig. 2:**
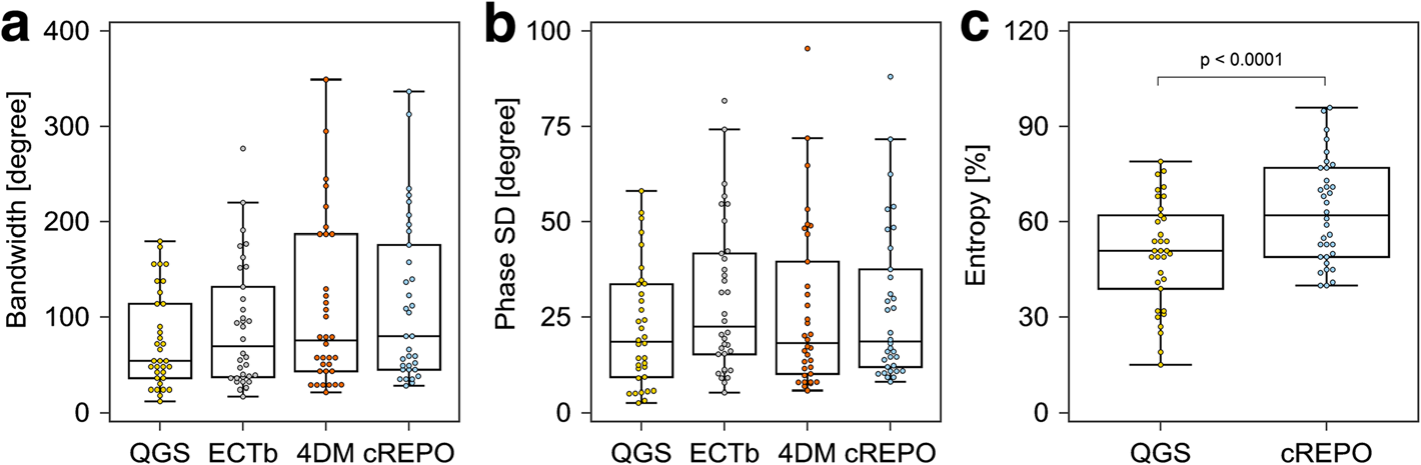
The box-and-whisker plots of bandwidth (**a**), phase SD (**b**), and entropy (**c**) in patients with suspected LV dyssynchrony (*n* = 34). These phase parameters were computed with QGS, ECTb, 4DM, and cREPO. *SD* standard deviation, *LV* left ventricular, *QGS* quantitative gated SPECT, *ECTb* Emory Cardiac Toolbox, *4DM* Corridor 4DM, *cREPO* cardioREPO

The phase distribution of patients with suspected LV dyssynchrony is shown in Fig. 2. There were no significant differences in bandwidth among the four software programs (QGS, 75.9° ± 50.3°; ECTb, 90.6° ± 65.1°; 4DM, 107.0° ± 86.3°; and cREPO, 114.0° ± 84.6°). There were also no significant differences in phase SD among the four software programs (QGS, 22.1° ± 15.6°; ECTb, 29.8° ± 20.0°; 4DM, 26.7° ± 21.7°; and cREPO, 27.7° ± 20.3°). Significant differences in entropy were observed between QGS and cREPO (50.0 ± 16.8% vs. 63.2 ± 16.2%, *p* < 0.0001).

### Correlation and agreement between software programs

**Fig. 3:**
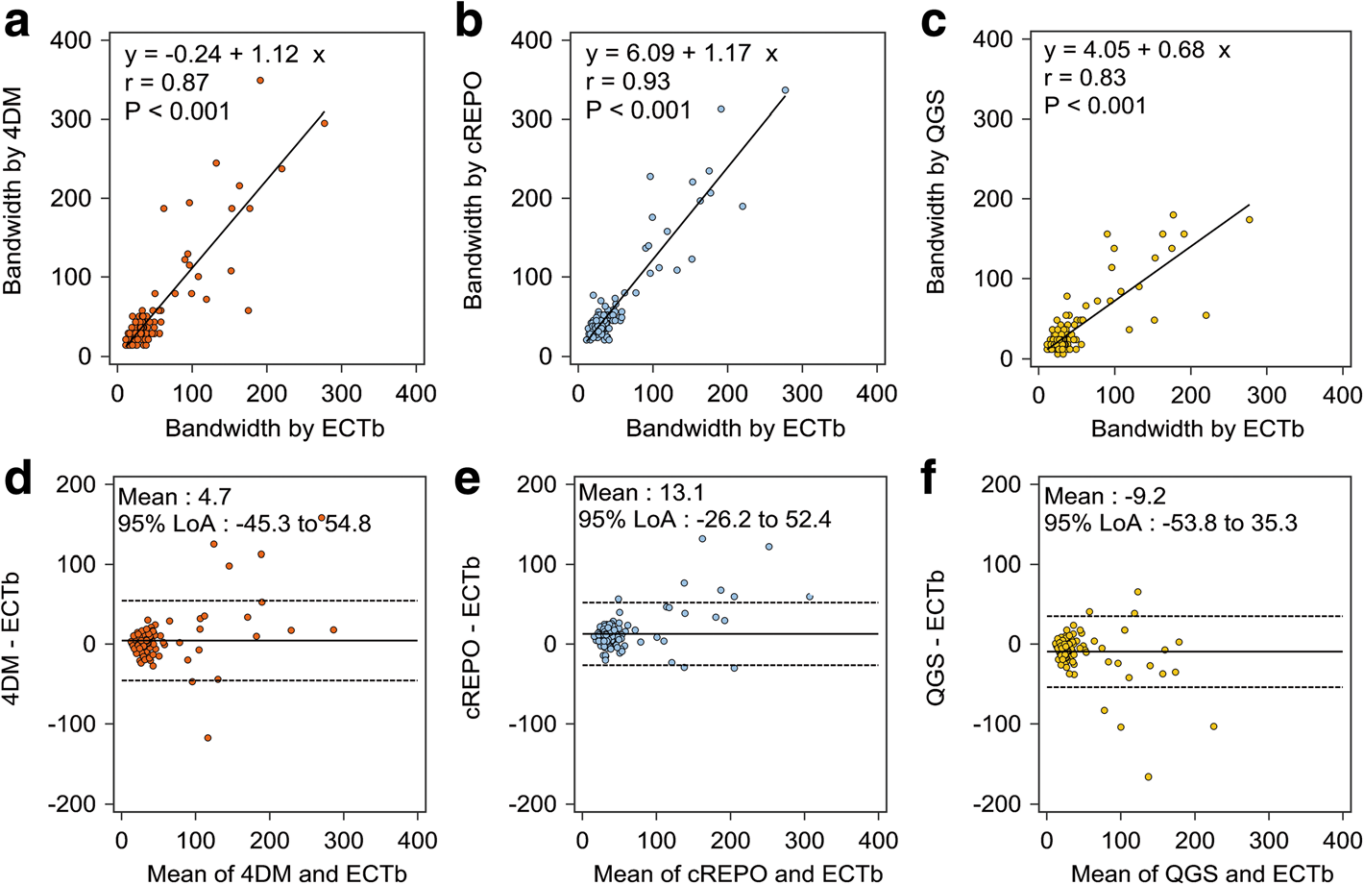
Scatter diagrams with regression line of bandwidth between 4DM and ECTb (**a**), cREPO and ECTb (**b**), and QGS and ECTb (**c**). The Bland-Altman plots of bandwidth between 4DM and ECTb (**d**), cREPO and ECTb (**e**), and QGS and ECTb (**f**). *Continuous lines* and *dashed lines* denote the mean difference between bandwidths by two software programs and upper and lower 95% limits of agreement, respectively. *QGS* quantitative gated SPECT, *ECTb* Emory Cardiac Toolbox, *4DM* Corridor 4DM, *cREPO* cardioREPO

The relationship and agreement among bandwidths obtained from the four software programs are shown in Fig. 3. The bandwidths obtained by ECTb was highly correlated with those obtained by 4DM (*r* = 0.87), cREPO (*r* = 0.93), and QGS (*r* = 0.83). The Bland-Altman analysis revealed that the mean differences were 4.7°, 13.1°, and −9.2° for 4DM vs. ECTb, cREPO vs. ECTb, and QGS vs. ECTb, respectively. The 95% limits of agreement (LoA) between cREPO and ECTb was narrower than those between 4DM and ECTb, and between QGS and ECTb.

**Fig. 4:**
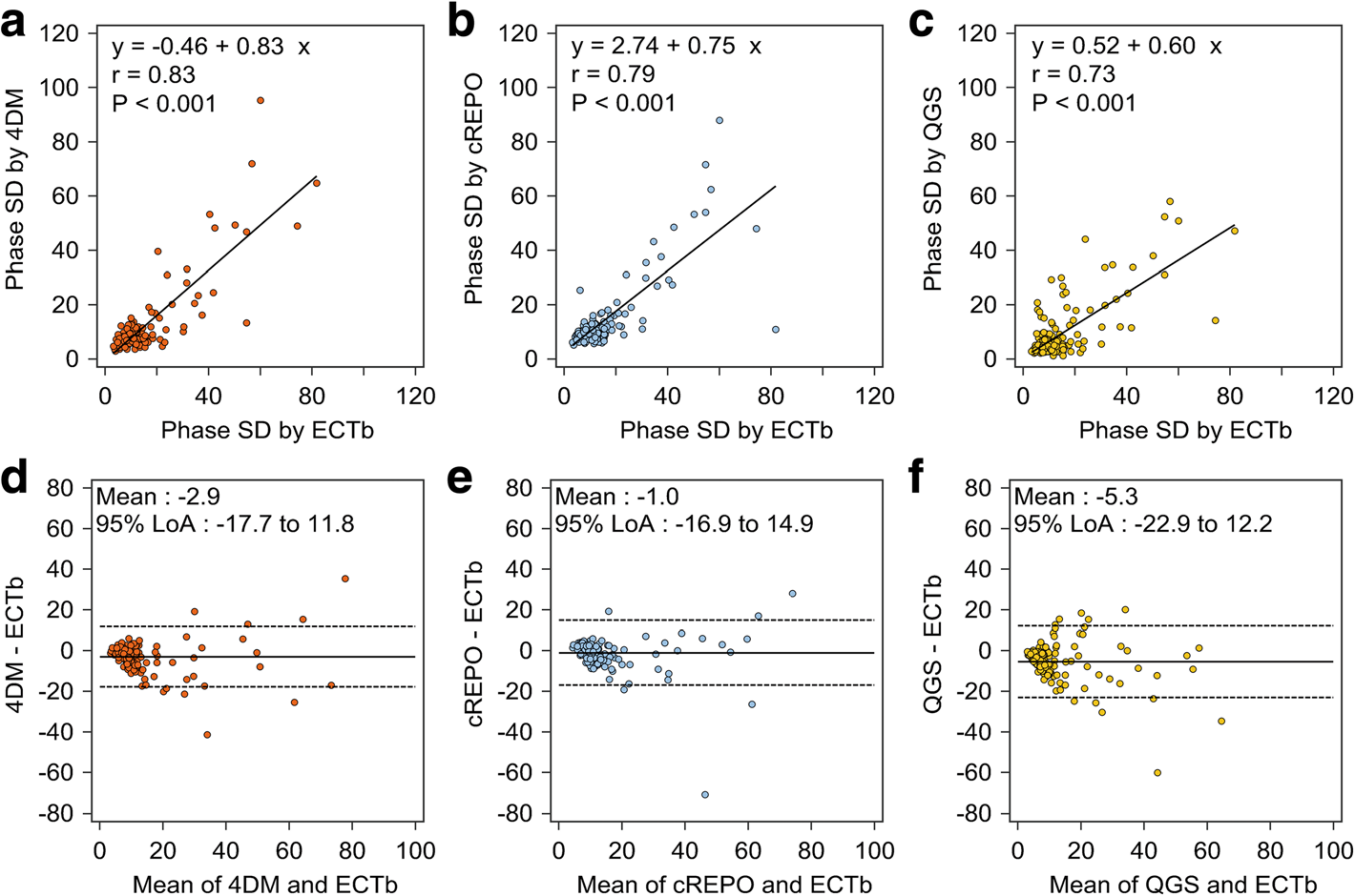
Scatter diagrams with regression lines of phase SD between 4DM and ECTb (**a**), cREPO and ECTb (**b**), and QGS and ECTb (**c**). The Bland-Altman plots of phase SD between 4DM and ECTb (**d**), cREPO and ECTb (**e**), and QGS and ECTb (**f**). *Continuous lines* and *dashed lines* denote the mean difference between phase SDs by two software programs and upper and lower 95% limits of agreement, respectively. *SD* standard deviation, *QGS* quantitative gated SPECT, *ECTb* Emory Cardiac Toolbox, *4DM* Corridor 4DM, *cREPO* cardioREPO

The relationship and agreement among phase SDs obtained from the four software programs are shown in Fig. 4. The phase SDs obtained by ECTb was highly correlated with those obtained by 4DM (*r* = 0.83), cREPO (*r* = 0.79), and QGS (*r* = 0.73). The Bland-Altman plots of the three software programs based on ECTb showed negative mean differences of −2.9°, −1.0°, and −5.3° for 4DM, cREPO, and QGS, respectively. The 95% LoA of QGS exhibited slightly wide variation in comparison with 4DM and cREPO.

**Fig. 5:**
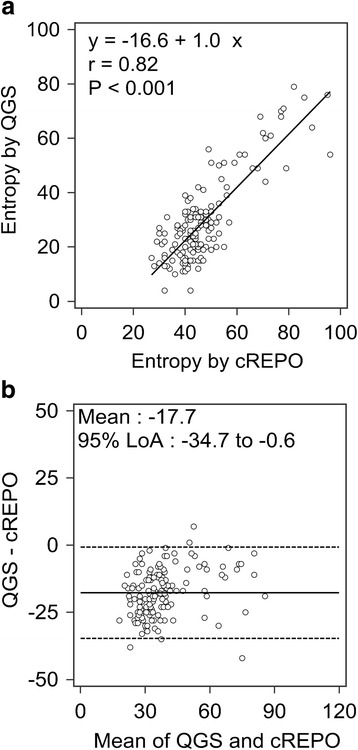
Scatter diagram with regression line of entropy between QGS and cREPO (**a**). The Bland-Altman plot of entropy between QGS and cREPO (**b**). *Continuous line* and *dashed lines* denote the mean difference between entropies by QGS and cREPO, and upper and lower 95% limits of agreement, respectively. *QGS* quantitative gated SPECT, *ECTb* Emory Cardiac Toolbox, *4DM* Corridor 4DM, *cREPO* cardioREPO

The relationship and agreement between entropies obtained from QGS and cREPO are shown in Fig. 5. Although the correlation coefficient was good (*r* = 0.82), the linear regression line was shifted to the right by 16.6%. The Bland-Altman analysis revealed that the mean of the difference was −17.7% and the 95% LoA ranged from −34.7 to −0.6%.

### ROC analysis

**Fig. 6:**
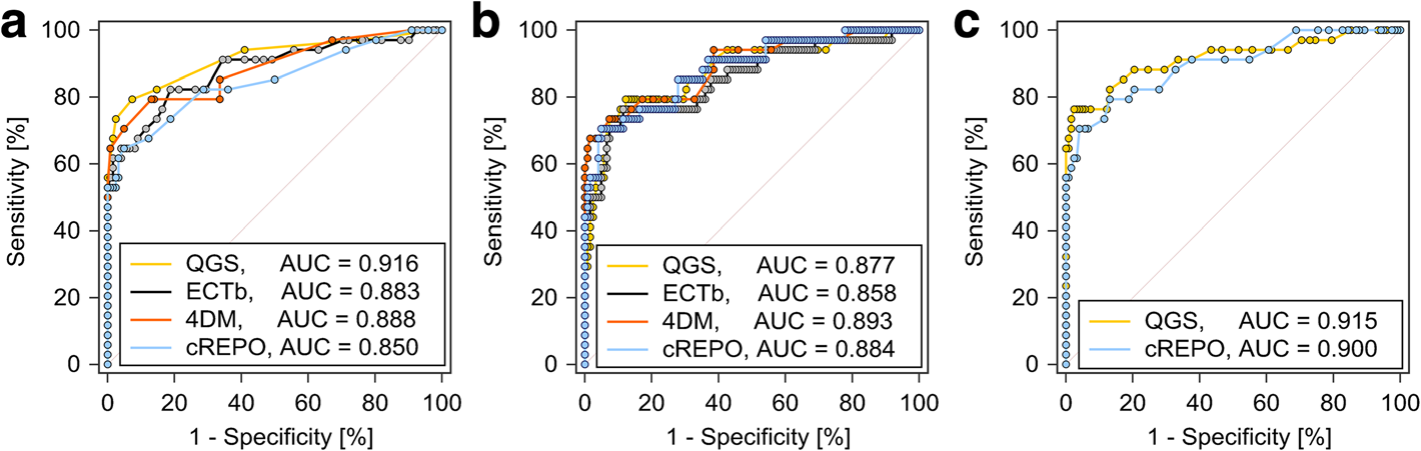
ROC curves of bandwidth (**a**), phase SD (**b**), and entropy (**c**) in QGS, ECTb, 4DM, and cREPO. *ROC* receiver operator characteristics, *SD* standard deviation, *AUC* area under the ROC curve, *QGS* quantitative gated SPECT, *ECTb* Emory Cardiac Toolbox, *4DM* Corridor 4DM, *cREPO* cardioREPO

**Fig. 7:**
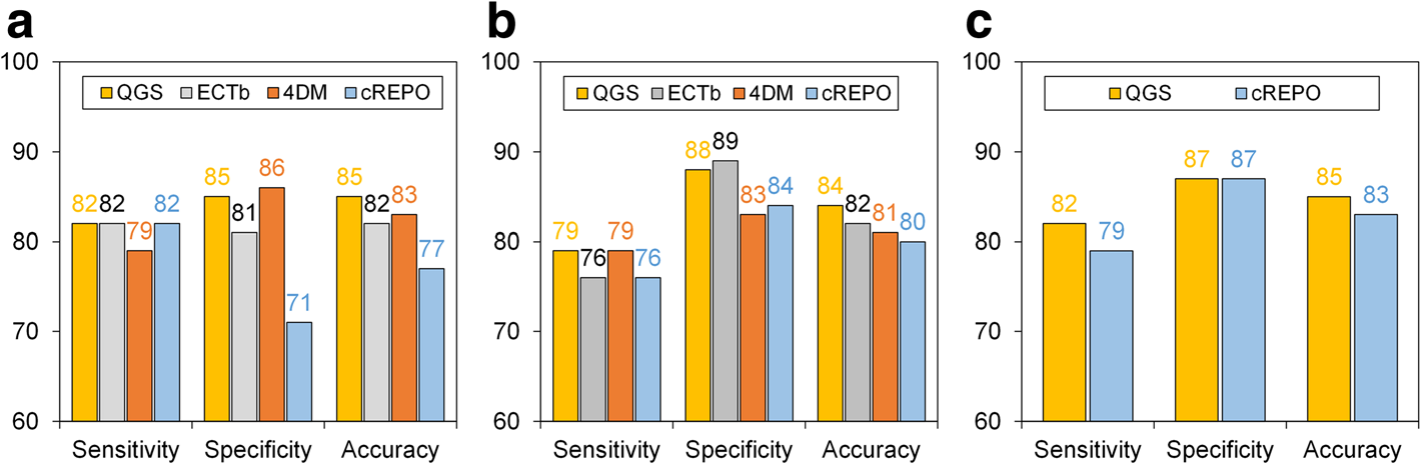
The sensitivity, specificity, and accuracy of ROC analysis in bandwidth (**a**), phase SD (**b**), and entropy (**c**). The sensitivity, specificity, and accuracy were computed by QGS, ECTb, 4DM, and cREPO. *ROC* receiver operator characteristics, *SD* standard deviation, *QGS* quantitative gated SPECT, *ECTb* Emory Cardiac Toolbox, *4DM* Corridor 4DM, *cREPO* cardioREPO

The ROC curve and its AUC are shown in Fig. 6. There were no significant AUC differences in bandwidths derived from the four software programs (QGS, 0.916 ± 0.033; ECTb, 0.883 ± 0.038; 4DM, 0.888 ± 0.038; and cREPO, 0.850 ± 0.044). The sensitivity, specificity, and accuracy are shown in Fig. 7. The sensitivity, specificity, and accuracy of the bandwidth ranged from 79 to 82%, 71 to 86%, and 77 to 85%, respectively. The lowest and highest cutoff points of bandwidth were 24° for QGS and 42° for cREPO, respectively, as observed in Table 2. When the optimal cutoff points of bandwidth for the four software programs were used, percentages of patients who were classified differently were 15% for 4DM, 28% for cREPO, 18% for ECTb, and 15% for QGS.

**Table 2:** Cutoff points for phase parameters in the four software programs

	QGS	ECTb	4DM	cREPO
Bandwidth (°)	24	35	36	42
Phase SD (°)	8.6	15.3	9.4	11.9
Entropy (%)	31	–	–	48

Abbreviations: *4DM* Corridor 4DM, *cREPO* cardioREPO, *ECTb* Emory Cardiac Toolbox, *QGS* quantitative gated SPECT

There were also no significant differences in AUC analysis in terms of the phase SD derived from the four software programs (QGS, 0.877 ± 0.039; ECTb, 0.858 ± 0.042; 4DM, 0.893 ± 0.035; and cREPO, 0.884 ± 0.036). The sensitivity, specificity, and accuracy of the phase SD ranged from 76 to 79%, 83 to 89%, and 80 to 84%, respectively. The lowest and highest cutoff points of phase SD were 8.6° for QGS and 15.3° for ECTb, respectively. The percentages of patients who were classified differently using cutoff points for phase SD were 18% for 4DM, 18% for cREPO, 21% for ECTb, and 14% for QGS.

No significant difference in AUC of entropy was observed between QGS (0.915 ± 0.034) and cREPO (0.900 ± 0.034). The sensitivity, specificity, and accuracy of an entropy were 82, 87, and 85% for QGS, and 79, 87, and 83% for cREPO, respectively. The cutoff point of entropy obtained by cREPO was higher than that obtained by QGS. The percentages of patients who were classified differently using cutoff points for entropy were 15% for cREPO and 14% for QGS.

The diagnostic performance of the bandwidth assessment was equivalent to that of the phase SD in 4DM, cREPO, and ECTb except for QGS (AUC; 0.916 vs. 0.877, *p* = 0.0047). Regarding the phase entropies obtained by QGS and cREPO, the ROC AUC of entropy showed significantly higher values than that of bandwidth in cREPO (*p* = 0.008) and that of phase SD in QGS (*p* = 0.016).

## Discussion

The main purpose of this study was to evaluate the diagnostic performance of four software packages: 4DM, cREPO, ECTb, and QGS in phase analysis. The relationship and agreement of phase parameters obtained from the four software programs were good. We could clearly determine that all software programs may be used reliably for phase analysis.

### Software algorithm for calculating LV dyssynchrony

Our results demonstrated that the cutoff values of bandwidth, phase SD, and entropy for evaluating LV dyssynchrony were variable depending on the software programs. The cause of these differences might be based on the delineation of the LV contour and the bin size of a phase histogram. Regarding the delineation of the LV contour, edge detection of the basal LV myocardium would play an important role in creation of the histogram for phase distribution. ECTb performs the function of excluding outliers of bandwidth and phase SD by smoothing the phase array data on a polar map. When the outliers of bandwidth and phase SD are observed in the aortic valve area or basal area of the polar map, we can manually exclude the aortic and basal areas from the phase calculation in cREPO. According to the bandwidth in Fig. 1a, the four software programs output discrete data of 6° for QGS, 1° for ECTb, 7° for 4DM, and 3° for cREPO. This result demonstrated that the bin sizes of a phase histogram differed in each software program.

### Normal phase distribution

With regard to normal phase distributions in previously reported studies, the mean bandwidth ranged from 27.9° to 42.0° and the mean phase SD ranged from 8.6° to 15.7° in ECTb [7, 25–28]. AIJaroudi et al. reported mean phase SDs of 6.1° in a stress condition and 10.2° at rest in 4DM [29]. QGS exhibited a mean bandwidth of 30.9°–80.2°, mean phase SD of 10°–22.2°, and mean entropy of 46.3–56.6% [23, 30]. Nakajima et al. have reported a mean bandwidth of 40°, mean phase SD of 10°, and mean entropy of 43% in cREPO. In comparison with our results, both mean bandwidth and phase SD calculated by QGS showed larger values in the reported studies.

### Which parameters are the best for phase analysis?

In analyses of phase distribution derived from GMPS, the 95% bandwidth, phase SD, entropy, kurtosis, and skewness are available using commercially software packages. Romeo-Farina reported that both bandwidth and phase SD showed excellent ROC AUCs, and these indices were the most important parameters [26]. ROC AUCs of bandwidth, phase SD, and entropy showed good diagnostic accuracy with values ≥0.850 in our results.

### Acquisition condition and gamma camera

The Japanese normal database consists of 122 normal patients enrolled from four hospitals. Various gamma cameras with LEHR and Vertex general-purpose (VXGP) collimators manufactured by ADAC, Elscint, Toshiba, and SIEMENS were used in the four hospitals. The acquisition conditions of myocardial perfusion SPECT were essentially the same in the four hospitals. All gated SPECT acquisitions were performed using 64 × 64 matrix with 16 frames per RR interval. However, 180 or 360° circular rotation of gamma cameras with step and shoot mode was used. When we calculated the normal bandwidth and phase SD using the ECTb software program derived from 180 or 360° SPECT acquisition in the four hospitals, there were no significant differences in bandwidth (30.0° ± 8.5° vs. 27.2° ± 9.3°; *p* = 0.11) and phase SD (11.6° ± 5.1° vs. 9.8° ± 4.5°; *p* = 0.07). Furthermore, when we additionally computed bandwidth and phase SD using the ECTb software program in each hospital, there were no significant differences among the four hospitals in bandwidths (30.9 ± 9.0, 30.0 ± 9.3, 29.0 ± 6.9, and 27.2 ± 9.3; *p* = 0.43 by analysis of variance (ANOVA)) and phase (10.4 ± 3.5, 11.8 ± 6.3, 12.4 ± 4.8, and 9.8 ± 4.5; *p* = 0.20 by ANOVA). Although there were no significant differences among four hospitals in normal bandwidth and phase SD, further clinical validation might be required to determine whether to harmonize the acquisition methodologies and gamma cameras in multicenter study or not.

### Limitation

We only performed phase analysis of rest GMPS in this study. Since myocardial perfusion count statistics vary under stress and rest conditions, ROC AUCs and cutoff values will be different under stress conditions. Furthermore, sex differences in bandwidth, phase SD, and entropy have been reported [7, 23, 30, 31]. The sex-specific cutoff values of phase parameters might clearly discriminate between normal patients and patients with suspected LV dyssynchrony. Moreover, further investigation would be required to determine the diagnostic accuracy of CRT using optimal cutoff values and to evaluate the effect of CRT on improvement of dyssynchrony in new follow-up study after the treatment. Although this study used a multicenter database in normal patients, patients with suspected LV dyssynchrony were enrolled in a single center; thus, multicenter validation should be conducted.

## Conclusions

The mean bandwidth, phase SD, and entropy significantly differed in 4DM, cREPO, ECTb, and QGS software programs in patients with normal perfusion and cardiac function. The LV dyssynchrony parameters of bandwidth, phase SD, and entropy obtained by 4DM, cREPO, and QGS were highly correlated with those by ECTb. Although the optimal cutoff values of bandwidth, phase SD, and entropy were variable depending on the software programs, the diagnostic performance by ROC analysis was equivalent. All four software programs can be used reliably for phase analysis in GMPS.

## Abbreviations

4DM: Corridor4DM
^99m^Tc MIBI: Technetium-99m methoxy-isobutyl-isonitrile
BBB: Bundle branch block
cREPO: CardioREPO
CRT: Cardiac resynchronization therapy
ECTb: Emory Cardiac Toolbox
HF: Heart failure
QGS: Quantitative gated SPECT
SPECT: Single-photon emission computed tomography
